# Prevalence of Migraines Among Medical Students in Saudi Arabia: A Systematic Review and Meta-Analysis

**DOI:** 10.7759/cureus.52086

**Published:** 2024-01-11

**Authors:** Abdulrahman M Albeshry, Fatimah S Alsaihati, Maha Mohammed Alsuwaiyan, Rawiyah Madani, Bushra Khamis Alanazi, Abdullateef A Allebdi

**Affiliations:** 1 Family and Community Medicine, Faculty of Medicine, University of Jeddah, Jeddah, SAU; 2 College of Medicine, Imam Abdulrahman Bin Faisal University, Dammam, SAU; 3 Radiology, King Saud Medical City, Riyadh, SAU; 4 Internal Medicine, Ibn Sina National College for Medical Studies, Jeddah, SAU; 5 Nephrology, North Medical Tower Hospital, Ministry of Health, Riyadh, SAU; 6 Preventive Medicine, Saudi Red Crescent Authority, Makkah, SAU

**Keywords:** awareness, meta-analysis, systematic review, saudi arabia, students, medical, headache, migraine, prevalence

## Abstract

Headaches are very common and often a common reason people visit emergency departments. Their prevalence among Saudi medical students was higher than the global average but aligned more closely with rates in certain countries. This regional variation may be attributed to factors such as academic pressures, lifestyle, and genetics. This systematic review and meta-analysis focused on assessing the prevalence of migraines among medical students in Saudi Arabia. Six cross-sectional studies were ultimately included in the meta-analysis, reporting a wide range of prevalence rates among Saudi medical students, from 5% to 26%. The pooled prevalence estimate was 23%, indicating a substantial burden of migraines among this population. The findings underscore the importance of tailored strategies and support systems within medical schools to address the impact of migraines on students' academic journey and overall well-being. Standardized diagnostic criteria and awareness programs are essential to effectively managing this condition among medical students. In conclusion, this study sheds light on the significant prevalence of migraines among medical students in Saudi Arabia, emphasizing the need for comprehensive management approaches and further research to refine prevalence estimates and develop targeted interventions.

## Introduction and background

Headaches are very common and often a reason people visit emergency departments, making up about 3% of these visits [1]. There are two main types of headaches: primary and secondary. Primary headaches are the usual kinds like tension headaches, migraines, and cluster headaches. Secondary headaches are more serious and can be caused by things like head injuries, problems with blood vessels in the brain, or nerve issues in the head [2]. The prevalence of migraine among medical students is particularly intriguing, given that it is the most common headache type in young adults [3]. Its prevalence ranges from 11% to 40% worldwide [4], with its frequency escalating during the educational years of study [5].

A migraine, a disorder with genetic influences, typically presents as moderate to severe episodes, often occurring on one side of the head and commonly accompanied by nausea, as well as heightened sensitivity to light and sound [2]. Globally, migraines impact around 12% of people, with a higher incidence in women (17%) compared to men (6%) annually [3]. As a major contributor to global disability, migraines accounted for 5.6% of all years lived with disability (YLDs), resulting in 45.1 million YLDs worldwide in 2016.

Clinical diagnosis of migraines is made based on specific criteria and subtypes [4]. Key diagnostic criteria include experiencing unilateral, pulsating, moderate to severe headaches with associated nausea and vomiting, typically in episodic patterns. These episodes can vary in duration from 4 to 72 hours, depending on the migraine subtype [2]. Several factors can exacerbate migraine episodes, including stress, anxiety, depression, intense physical activity, negative thinking patterns, hormonal fluctuations during menstruation, and dietary habits [2,4].

In medical students, anxiety and stress, largely due to rigorous academic challenges and significant life changes, are the most frequent triggers for migraine episodes [5,6]. These can detrimentally impact their academic performance. Previous research indicates that migraine prevalence among medical students ranges from 12% to 28% [7-10], highlighting a significant issue that can affect their quality of life, work efficiency, and educational outcomes.

The pursuit of medical education in Saudi Arabia, characterized by rigorous academic demands and clinical responsibilities, presents a potential risk factor for the development or aggravation of migraines [11]. This systematic review and meta-analysis aim to synthesize existing research to provide a clear picture of the prevalence of migraines among medical students in Saudi Arabia.

## Review

Materials and methods

In order to comprehensively gather studies relevant to our topic, we designed an extensive search strategy. This included exploring key databases like PubMed, Scopus, Web of Science, and the Cochrane Library. We focused on literature published up to the current year to include the most up-to-date research available. Our search was structured using a combination of Medical Subject Headings (MeSH) terms and free-text words, aiming to thoroughly capture pertinent articles. The primary search terms we used were “migraine,” “headache,” “medical students,” and “Saudi Arabia.” These terms were interconnected using the Boolean operators “AND” and “OR” to include a range of related keywords and expressions. For instance, our search string in PubMed was as follows: (“migraine” OR “headache”) AND “medical students” AND “Saudi Arabia.”

To broaden our scope and ensure no relevant study was overlooked, we extended our search to include regional databases such as the Saudi Medical Literature Database and the Index Medicus for the Eastern Mediterranean Region. Additionally, we manually reviewed the reference lists of identified articles and related review papers to discover any further studies that may not have been captured in our initial database search.

Inclusion and Exclusion Criteria

In our systematic review and meta-analysis, we applied specific inclusion and exclusion criteria to ensure the studies' relevance and quality of reporting data, focusing exclusively on cross-sectional studies. We included cross-sectional studies that examined the prevalence of migraines among medical students in Saudi Arabia. These studies were required to provide detailed data on the prevalence or incidence of migraines, diagnosed according to accepted medical standards, such as the International Classification of Headache Disorders. We limited our scope to studies conducted within Saudi Arabia and published from 2000 to 2023. The language of the studies was restricted to English or Arabic to align with our review capabilities.

On the exclusion side, we omitted any studies that were not cross-sectional in design, including clinical trials, reviews, editorials, and case reports. We also excluded studies focusing on populations other than medical students in Saudi Arabia or those involving mixed populations where specific data for medical students couldn't be distinctly extracted. Research not directly measuring the prevalence or incidence of migraines or employing non-standard diagnostic criteria was not considered. Additionally, studies conducted outside Saudi Arabia, those with incomplete data or lacking essential methodological details, and those published in languages other than English or Arabic were excluded. This stringent selection process was aimed to ensure our review comprehensively and accurately reflected the prevalence of migraines among medical students in Saudi Arabia.

Study Selection

Our selection methodology for studies was rigorously structured to ensure exhaustive identification and evaluation. Initially, all identified articles were imported into EndNote, facilitating duplicate removal. The first screening phase involved two independent reviewers meticulously assessing study titles to eliminate irrelevant or non-compliant studies. This was followed by a detailed review of abstracts by these reviewers to refine our selection further.

Articles deemed relevant at this stage underwent a full-text review by another pair of independent reviewers to confirm eligibility against our criteria. This comprehensive evaluation ensured a thorough assessment of methodologies, populations, and outcomes. Discrepancies at any stage were resolved through discussion, or by consulting a third reviewer for a conclusive decision. Additionally, a manual search of references in selected studies was conducted to ensure the inclusion of all relevant literature.

Data Collection

In our systematic review and meta-analysis, we conducted a detailed data collection process focusing on the prevalence of migraines among medical students in Saudi Arabia. The data was extracted from various cross-sectional studies, and each study was scrutinized for specific information. This included the identification of the primary author and the year of publication, ensuring including all relevant studies published from 2000 to 2023. We noted the gender of the study participants, which in all cases included both male and female students. The specific universities where these studies were conducted were recorded, providing insights into geographical and educational contexts.

Additionally, we collected data on the age groups or academic years of the students involved in each study, ranging from the first year to the final year of medical school. The total number of participants in each study was also documented, offering a sense of the study's scale. Crucial to our analysis was the criteria or methods used in these studies to diagnose migraines, which varied from the ID Migraine™ test to more comprehensive questionnaires like the Headache Intake Questionnaire from the Cleveland Clinic. Finally, we recorded the prevalence rates of migraines as reported in each study.

Quality Assessment

In our systematic review, assessing the quality of the included studies was a crucial step to ensure their methodological soundness and identify any potential biases. We utilized a recognized tool specifically designed for evaluating observational studies. The chosen tool, the Newcastle-Ottawa Scale (NOS), is known for its detailed criteria that evaluate studies from three key aspects: the selection of study groups, the comparability of these groups, and the determination of the outcome of interest in case-control or cohort studies [12].

Two independent reviewers conducted the evaluation of each study using the NOS criteria. This process involved scoring each study based on the quality of its selection, comparability, and exposure or outcome measures. The resulting scores were then utilized to classify the studies into three levels of quality: high, moderate, or low. Studies that adhered closely to most of the NOS criteria were deemed high-quality, indicating a lower likelihood of bias. Those that met some criteria were considered moderate quality, while studies failing to meet several criteria were categorized as low-quality, reflecting a higher potential for bias. The independent evaluations by the reviewers were essential for an unbiased and comprehensive assessment. Any discrepancies in quality scores between the reviewers were resolved through discussion until a consensus was reached.

Statistical Analysis

In the process of managing and analyzing data for our study on the prevalence of migraines among medical students in Saudi Arabia, we employed Rayyan - Qatar Computing Research Institute (QCRI) as a valuable tool for efficient article management [13]. This approach ensured systematic organization and the selection of relevant studies, contributing to the reliability of our analysis. The statistical analysis was conducted using STATA version 17 (StataCorp LP, College Station, TX), chosen for its advanced capabilities in handling complex meta-analytical data. Our meta-analysis was focused on determining the prevalence of migraines among medical students. To achieve this, we applied a random-effects model based on the weighted inverse variance method. This model was selected to account for the expected high heterogeneity inherent in studies of this nature, given variations in study designs, student populations, and diagnostic criteria. The outcomes of our analysis were visually presented using forest plots, which provide a clear representation of pooled proportions and their corresponding 95% confidence intervals (CIs), aiding in the interpretation of our results. To assess the degree of variability and the robustness of our findings, we utilized the Higgins I² test statistic. An I² value exceeding 50% was considered indicative of substantial heterogeneity, a common feature in migraine prevalence studies.

Results

Article Screening and Selection

**Figure 1:**
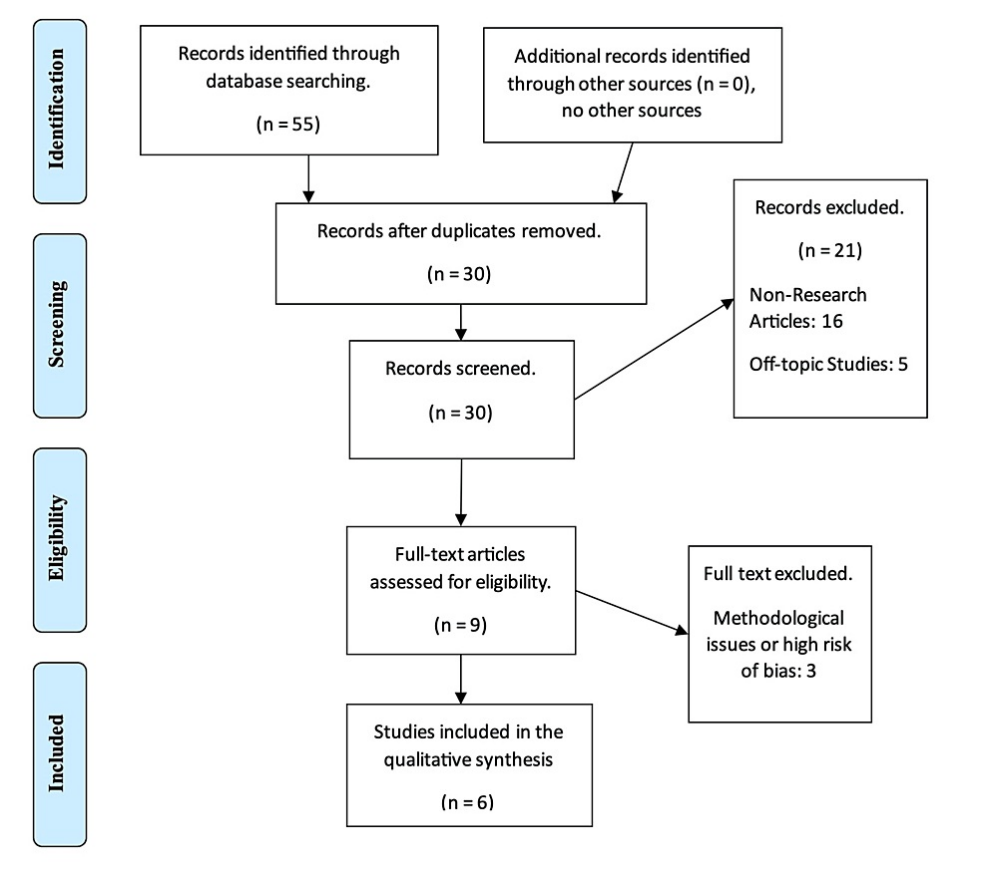
Flow diagram of article screening and selection process for studies.

The initial phase of our systematic review involved a comprehensive search that resulted in 55 potential articles. After deduplication, 30 unique articles remained for evaluation. Rigorous screening led to the exclusion of 21 articles due to reasons like irrelevance, non-research related content, or deviation from the topic. A thorough full-text review of the remaining nine articles was then carried out, ultimately selecting six studies that met our strict inclusion criteria, ensuring their quality and relevance for the meta-analysis as displayed in Figure 1.

Migraine Prevalence Among Medical Students in Saudi Arabia

**Figure 2:**
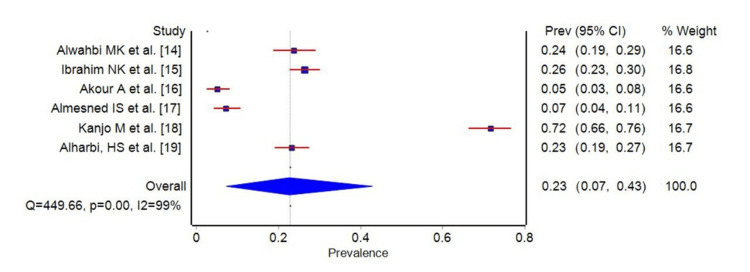
Forest plot of the prevalence of migraines among medical students. Blue square boxes represent the pooled prevalence

In our systematic review and meta-analysis, we included six studies in the final analysis. The individual studies reported prevalence rates ranging from 5% to 26%, reflecting notable variation. The pooled prevalence of migraines across all studies was found to be 23%. However, the CIs were wide, spanning from 7% to 43%, indicating considerable uncertainty around the true effect size as shown in Figure 2.

The statistical analysis revealed substantial heterogeneity, with an I-squared (I²) value of 99%, indicating that 99% of the variability in prevalence estimates could be attributed to heterogeneity rather than chance. This is supported by Cochran's Q statistic of 449.66, which is associated with a p-value of less than 0.001, suggesting that the observed heterogeneity is statistically significant. The tau-squared (tau²) statistic was calculated at 0.26, further quantifying the between-study variance.

Characteristics of Included Studies

Our meta-analysis reviewed six cross-sectional studies on the prevalence of migraines among Saudi Arabian medical students [14-19], spanning 2017 to 2023. Table 1 shows studies incorporated a mix of genders and ranged from first-year students to those in their sixth year. Alwahbi et al. (2017) and Ibrahim et al. (2017) reported prevalence rates of 23.7% and 26.3% at King Saud bin Abdulaziz and King Abdulaziz Universities, respectively, using the ID Migraine™ test. Akour et al. (2018) found a 5% prevalence at Jazan University using IHS criteria, while Almesned et al. (2018) reported 7.1% prevalence at King Saud bin Abdulaziz University using a specialized questionnaire. Kanjo et al. (2021) observed a high prevalence of 71.6% at Fakeeh College, and Alharbi et al. (2021) reported a 23.2% prevalence at Umm-Al-Qura University, both utilizing the ID Migraine™ test and the MSQ-5 respectively. The results underscore the variable burden of migraines across different universities.

**Table 1:** Characteristics of included studies.

Author	Year	Study Type	Gender	University	Age group	Participants	Criteria used to diagnose Migraine	Prevalence
Alwahbi et al. [14]	2017	Cross-sectional	Both	King Saud bin Abdulaziz University	From the first year till the fifth year of the college	270	ID Migraine™ test	23.7%
Ibrahim et al. [15]	2017	Cross-sectional	Both	King Abdulaziz University	From the second year till the fifth year of the college	566	ID Migraine™ test, Numeric Pain Rating Scale (NPRS) for severity assessment	26.3%
Akour et al. [16]	2018	Cross-sectional	Both	Jazan University	19-25 years	258	IHS criteria	5%
Almesned et al. [17]	2018	Cross-sectional	Both	King Saud bin Abdulaziz University	Third- and fourth year	264	Headache Intake Questionnaire of the Cleveland Clinic, Toronto, Ontario, Canada	7.1%
Kanjo et al. [18]	2021	Cross-sectional	Both	Fakeeh College	All academic years	313	ID Migraine™ test	71.6%
Alharbi et al. [19]	2021	Cross-sectional	Both	Umm-Al-Qura University	From second year to sixth year	406	Migraine Screen Questionnaire (MSQ-5)	23.2%

Quality Assessment of Included Studies

Table 2 provides a quality assessment of the studies included in our analysis, evaluating their methodology based on selection, comparability, and exposure/outcome, with a total possible score of 9 points. In the quality assessment of the included studies, four were rated as high quality and two as moderate quality. Alwahbi et al. (2017) and Kanjo et al. (2021) both scored 7 points, while Ibrahim et al. (2017) and Alharbi et al. (2021) scored higher with 8 points, all classified as high quality. Akour et al. (2018) and Almesned et al. (2018) each scored 6 points, falling into the moderate quality category. The assessment was based on selection, comparability, and exposure/outcome, with a maximum of 9 points.

**Table 2:** Quality assessment of included studies.

Study (Author, Year)	Selection (Max 4 Points)	Comparability (Max 2 Points)	Exposure/Outcome (Max 3 Points)	Total Score (Max 9 Points)	Quality Tier
Alwahbi et al., 2017 [14]	3	2	2	7	High Quality
Ibrahim et al., 2017 [15]	4	2	2	8	High Quality
Akour et al., 2018 [16]	2	2	2	6	Moderate Quality
Almesned et al., 2018 [17]	2	2	2	6	Moderate Quality
Kanjo et al., 2021 [18]	3	2	2	7	High Quality
Alharbi et al., 2021 [19]	4	2	2	8	High Quality

Discussion

This systematic review and meta-analysis critically evaluated the prevalence of migraines among medical students in Saudi Arabia. The prevalence of migraines among medical students in Saudi Arabia, as highlighted in our meta-analysis, shows interesting contrasts and similarities when compared to other studies conducted among medical students globally.

In our analysis, Saudi medical students exhibited a wide range of prevalence rates, from 5% to 71.6%, with a pooled prevalence of 23%. This rate is higher than the 17.27% observed among Egyptian medical students, indicating regional variations even within the Middle East [20]. However, it aligns more closely with the 21.4% prevalence found in Bangladesh [21].

Notably, the prevalence in Saudi Arabia is lower than the substantial 42% reported in Penang state, where over 61.8% of those experiencing headaches met the IHS criteria for migraines [22]. This indicates that the issue might be more acute in certain regions, perhaps due to varying educational pressures, lifestyle factors, or genetic predispositions [23].

In Kuwait, 27.9% of medical students were suggested to have migraines based on the ID-Migraine™, a rate that is quite similar to the higher end of prevalence in Saudi Arabia [24]. These comparisons reveal that while migraines are a common concern among medical students globally, the extent of their impact varies significantly from one region to another. This variability could be influenced by different academic environments, cultural factors, and healthcare access, which warrants tailored strategies in each region to effectively address and manage migraines among medical students.

The prevalence of migraines among medical students in Saudi Arabia is markedly higher than the general global prevalence of 14% [1]. This contrast may reflect the unique pressures faced by medical students, such as intense academic demands and related stress factors. When compared to general populations in other regions, Saudi medical students experience higher prevalence rates than those reported in the USA (11.7%) [25], Germany (10.6%) [26], and even the UK (14.3%) [27]. However, the rates are more comparable to those observed in India (22.8%) [28] and Turkey (16.4%) [29].

The findings call for a comprehensive approach to migraine management in medical schools, including better diagnostic procedures, awareness programs, and support systems. Policies should be aimed at creating a supportive environment that accommodates students suffering from migraines.

Limitations

Our analysis is not without limitations. The exclusion of non-English articles and grey literature could omit relevant data. The variability in diagnostic criteria across studies introduces methodological heterogeneity, complicating the synthesis of results. Moreover, the cross-sectional nature of the included studies precludes causality inference.

## Conclusions

Migraines are a significant concern among medical students in Saudi Arabia, with a high degree of variability in reported prevalence. The findings emphasize the need for standardized diagnostic criteria and tailored interventions to manage this condition effectively. High-quality studies with uniform methodologies are essential for accurate prevalence estimates and for developing strategies to mitigate the impact of migraines on students’ academic journey and overall wellbeing. The findings call for a comprehensive approach to migraine management in medical schools, including better diagnostic procedures, awareness programs, and support systems. Policies should be aimed at creating a supportive environment that accommodates students suffering from migraines.
